# Calcium Phosphate Product Is Associated with Subclinical Carotid Atherosclerosis in Type 2 Diabetes

**DOI:** 10.1155/2017/3498368

**Published:** 2017-08-03

**Authors:** Anna Ramírez-Morros, Minerva Granado-Casas, Nuria Alcubierre, Montserrat Martinez-Alonso, Jordi Real, Esmeralda Castelblanco, Aureli Esquerda, Gonzalo Cao, Esther Rubinat, Marta Hernández, Núria Alonso, Elvira Fernández, Didac Mauricio

**Affiliations:** ^1^Health Sciences Research Institute Germans Trias i Pujol, Camí de les Escoles S/N, 08916 Badalona, Spain; ^2^Department of Endocrinology and Nutrition, Health Sciences Research Institute and University Hospital Germans Trias i Pujol, Carretera Canyet S/N, 08916 Badalona, Spain; ^3^Biomedical Research Institute of Lleida, University of Lleida, Rovira Roure 80, 25198 Lleida, Spain; ^4^Biostatistics and Epidemiology Unit, Biomedical Research Institute of Lleida, University of Lleida, Rovira Roure 80, 25198 Lleida, Spain; ^5^CIBER of Diabetes and Associated Metabolic Diseases (CIBERDEM), Instituto de Salud Carlos III, Madrid, Spain; ^6^Epidemiology and Public Health, Universitat Internacional de Catalunya, Sant Cugat, Spain; ^7^Unitat de Suport a la Recerca Barcelona, Institut Universitari d'Investigació en Atenció Primària Jordi Gol (IDIAP Jordi Gol), Barcelona, Spain; ^8^Department of Laboratory Medicine, University Hospital Arnau de Vilanova, Rovira Roure 80, 25198 Lleida, Spain; ^9^Department of Endocrinology and Nutrition, University Hospital Arnau de Vilanova, Rovira Roure 80, 25198 Lleida, Spain; ^10^Unitat de Deteccio i Tractament de Malalties Aterotrombotiques, University Hospital Arnau de Vilanova, Lleida, Spain; ^11^Department of Nephrology, University Hospital Arnau de Vilanova, Lleida, Spain

## Abstract

**Aims:**

To assess whether circulating 25-hydroxyvitamin D_3_ (25OHD) and mineral metabolism-related factors (serum phosphate, calcium, and parathormone) are associated with subclinical carotid atherosclerosis (SCA), defined as the presence of carotid atherosclerotic plaques (main study outcome), in patients with type 2 diabetes mellitus (T2DM) without kidney disease or previous cardiovascular disease.

**Methods:**

We undertook a post hoc analysis of a cross-sectional study in adults with T2DM in whom we evaluated SCA. A total of 303 subjects with T2DM were included. Clinical variables and carotid ultrasound imaging were obtained.

**Results:**

We found no association of 25OHD with the presence of SCA. However, calcium phosphate (CaP; mg^2^/dL^2^) product was positively associated with the presence of carotid plaques (OR_adj_ = 1.078; 95% CI: 1.017–1.142). An inverse association was observed between higher levels of 25OHD (≥30 ng/mL versus <20 ng/mL concentrations) and common carotid intima-media thickness (cIMT; mm) (*β*_adj_ ± SE = −0.055 ± 0.024). We conclude that the CaP product is independently associated with the presence of established subclinical carotid atherosclerosis in patients with T2DM.

## 1. Introduction

Patients with type 2 diabetes mellitus (T2DM) are at 2- to 4-fold higher risk of cardiovascular mortality compared with nondiabetic subjects [1]. Further, type 2 diabetic subjects will lose 16–18 quality-adjusted life-years due to diabetes and will die approximately 6 years earlier than the nondiabetic population [2, 3]. T2DM and atherosclerosis share almost all the traditionally recognized main common risk factors: obesity, high blood pressure, dyslipidemia, dietary factors, sedentary lifestyle, and age. Diabetic atherosclerosis develops earlier and has a more extensive and diffuse presentation than atherosclerosis in nondiabetic subjects [4]. Early lesions in several arterial territories (i.e., plaques) can be identified in atherosclerosis. There is an increased burden of atherosclerotic plaques in patients with T2DM well before the appearance of cardiovascular events [5, 6]. Further, the presence of carotid plaques is a stronger predictor of future events than carotid intima-media thickness [7]. Additionally, in type 2 diabetic subjects, the presence of carotid plaques strongly predicts future cardiovascular events [6]. Nevertheless, clinical prediction of cardiovascular disease in type 2 diabetes is still an important challenge. Unfortunately, additional biomarkers for improving cardiovascular risk prediction have not yet been proved to be helpful in the clinical setting [8]. Thus, the identification of new biomarkers applicable to clinical practice is a current challenge to improve risk evaluation and clinical outcomes.

The contribution of different factors involved in the regulation of mineral metabolism in the pathogenesis of atherosclerosis and subsequent cardiovascular events is supported by experimental and clinical evidence [9–11]. Among these factors, vitamin D is probably the most extensively studied one. Vitamin D is a hormone that is instrumental in maintaining mineral homeostasis, bone mineralization, and regulation of parathyroid hormone secretion [9]. Although its active form is 1,25-hydroxyvitamin D_3_, the circulating concentrations of 25-hydroxyvitamin D_3_ (25OHD) is the parameter that indicates the vitamin D deficiency/sufficiency status. Evidence from some association studies pointed to an association of vitamin D deficiency with subclinical atherosclerosis or established cardiovascular disease [10, 12, 13]. Also, cohort studies found that lower vitamin D levels were predictive of cardiovascular morbidity and mortality [14]. However, in other recent well-designed cohort studies, vitamin D was not found to be associated with subclinical atherosclerosis and its progression [15].

Although calcium and phosphate homeostasis is tightly regulated by vitamin D and parathormone (PTH), less attention has been paid to their potential involvement in the pathogenesis of atherosclerosis or as predictors of cardiovascular disease. Elevated concentrations of calcium and phosphate have also been related to the development of atherosclerosis and cardiovascular events [16–22]. However, not all studies addressing the role of vitamin D in the development of atherosclerosis included simultaneously the determination of calcium and phosphate or their inclusion in their prediction or association models.

Considering all the evidence described above, we hypothesized that vitamin D and other markers of mineral metabolism are associated with the presence of carotid atherosclerotic plaques in patients with T2DM free from established chronic kidney disease and cardiovascular disease. Thus, the aim of this study was to test whether circulating vitamin D and other mineral metabolism-related factors, that is, calcium, phosphate, and intact parathormone, are associated with the presence of subclinical carotid atherosclerosis in type 2 diabetes mellitus patients.

## 2. Methods

### 2.1. Study Design and Subjects

This is an ancillary study of a previous cross-sectional study in adult type 2 diabetic patients recruited at a single reference hospital in Catalonia, Spain. The primary objective of the current approach was to assess the potential association between subclinical atherosclerosis and 25OHD and other mineral metabolism factors: calcium, phosphate, and PTH. The study population has been recently described in the previous study that was designed to assess subclinical carotid atherosclerosis and cerebral microvascular disease in patients with T2DM [5, 23]. A total of 303 subjects with (*n* = 180) or without (*n* = 123) subclinical atherosclerosis were included on the basis of availability of serum samples. The inclusion criteria were age from 40 to 75 years old, normal renal function (estimated glomerular filtration rate (eGFR) > 60 mL/min), and availability of the main study variables to perform the study assessments. The variables that were considered for this substudy were weight, height, waist, smoking habit, presence of dyslipidemia and hypertension, duration of diabetes and its treatment, lipid profile, age, sex, ethnicity, and the carotid ultrasound parameters.

The exclusion criteria were defined as follows: established chronic kidney disease (defined as either albumin/creatinine > 300 mg/g and/or eGFR < 60 mL/min), previous supplementation with vitamin D and/or calcium, and previously known cardiovascular disease events or associated revascularization procedures, including coronary heart disease, cerebrovascular disease, or peripheral vascular disease (this included the diagnosis of diabetic foot disease). For this purpose, the patients' clinical records were thoroughly reviewed in addition to the anamnestic evaluation and physical examination.

A detailed description of the study subjects and clinical procedures is provided elsewhere [5]. Out of 312 subjects of the original study, 9 participants were finally excluded: 4 patients were taking oral vitamin D and/or calcium supplements, and 5 other patients had no samples for the determination the main lab study variables. A total of 18 patients did not have data on physical activity and dietary intake questionnaires. The study protocol was approved by the Ethics Committee of University Hospital Arnau de Vilanova, and all participants signed a written consent form.

### 2.2. Clinical and Laboratory Procedures

Relevant demographic and clinical data were obtained from each subject. Weight, height, and waist circumference were measured using standardized clinical procedures. Patients were classified as having hypertension or dyslipidemia when they were receiving antihypertensive or lipid-lowering treatment, respectively. Laboratory variables were determined in fasting blood and spot urine samples. All analytical tests were performed using standard laboratory methods. Basic blood and urine biochemistry were determined using a Hitachi Modular DDPP analyzer (Roche Diagnostics, Indianapolis, USA). Hemoglobin A1c was measured using an HPLC Variant II Turbo (Bio-RAD, Hercules, USA). Intact parathormone was measured in an Elecsys E170 analyzer (Roche Diagnostics, Indianapolis, USA) by electrochemiluminescence immunoassay with 2.2% and 6% intra- and interassay variability, respectively. Serum 25OHD concentrations were measured using an Architect i2000SR analyzer (Abbott Diagnostics, Lake Forest, USA) by a chemiluminescent microparticle immunoassay with an intra- and interassay variability values of 2.3% and 6.2%, respectively. Vitamin D deficiency was defined as a concentration of 25OHD below 20 ng/mL, and vitamin D insufficiency as values between 20 and <30 ng/mL, as defined by the Endocrine Society [24]. These thresholds were used also for the analyses of the study. Dietary intake of vitamin D and calcium was evaluated using a 101-food-item semiquantitative food frequency validated questionnaire (available at http://bibliodieta.umh.es/files/2011/07/CFA101.pdf) [25], and the dietary content of vitamin D and calcium was calculated using the food composition tables of the US Department of Agriculture and supplemented with Spanish sources [25, 26]. The season of assessment was classified according to the following distribution: winter (January–March), spring (April–June), summer (July–September), and autumn (October–December), according to the date of blood sampling. Physical activity was categorized as sedentary and active defining sedentary subjects as those expending less than 10% of their daily energy expenditure in activities using 4 or more basal metabolism rate multiples (equal or greater physical activity expenditure than brisk walking for 30 minutes) [27].

### 2.3. Carotid Ultrasound Imaging Procedures

Subclinical carotid atherosclerosis (SCA) was defined as the presence of any atherosclerotic plaque in the carotid arteries. All participants underwent the same carotid ultrasound protocol performed by the same researcher, who was blinded to the characteristics of the study participants. Ultrasound images were obtained with a Siemens Sequoia 512 and a 15 MHz linear array probe. To identify the presence of plaques a 2D-mode and colour Doppler imaging, techniques were likewise used in longitudinal and transverse planes. The common carotid intima-media thickness (cIMT) was determined using conventional ultrasound procedures as measured from the media-adventitia interface to the intima-lumen interface. The intraobserver variability of the IMT image reading, calculated as the intraclass correlation coefficient, of the researcher performing this procedure is 0.93 (95% confidence interval: 0.89–0.98). According to the Mannheim consensus [28], plaque was defined as a focal structure encroaching into the arterial lumen at least 0.5 mm or 50% of the surrounding cIMT value or that demonstrates a thickness > 1.5 mm. A detailed description of the ultrasound procedures used in this study has been recently described [5, 29].

### 2.4. Statistical Analysis

Quantitative variables were summarized using mean and standard deviation for those normally distributed (according to Shapiro-Wilks test) and as median and interquartile intervals otherwise. Qualitative variables were summarized using absolute and relative frequencies. For the variables showing a nonlinear relationship with any specific study outcome, some recoding options were tested. The relationship between potential risk factors and subclinical atherosclerosis was analyzed by adjusting multivariable logistic regression models for the presence of any plaque and by adjusting multivariable linear regression models for the common intima-media thickness. Logistic regression model building process was started by a bivariate analysis consisting of the chi-squared or McNemar tests that were used to compare qualitative variables and the *t*-test or Mann–Whitney *U* test that were used to compare quantitative variables (normally distributed or not, resp.) between two groups. A first multivariable logistic regression model was adjusted with all the clinically significant variables. The model was reduced by dropping one variable in each step, starting by the one with the highest nonsignificant *p*-value according to the likelihood ratio test (LRT). Calibration and discrimination for the final proposed logistic regression models were assessed by the Hosmer-Lemeshow goodness-of-fit test and the AUC. For the final linear regression model, the Kolmogorov-Smirnov goodness-of-fit test was performed and the coefficient of determination was provided. Adjusted association measures were the odds ratio (OR) and their 95% confidence interval (95% CI) with logistic regression models and effect (*β*) and standard error (±SE) with linear regression. The open source statistical analysis software package R [30] and a statistical significance level of *p* < 0.05 were used.

## 3. Results

Clinical and biochemical characteristics of patients with T2DM according to their SCA status are shown in Table 1. In the group of subjects with dyslipidemia, 130 (92.9%) patients were receiving treatment with statins (84 with and 46 without carotid plaques). Among patients with carotid plaques (i.e., SCA), 81 had only 1 plaque and 99 had 2 or more carotid plaques. As expected, subjects with SCA showed also increased values of common carotid intima-media thickness, which is an early measure of subclinical carotid atherosclerosis (0.83 versus 0.73 mm; *p* < 0.001). Subjects with SCA were also older than those without SCA (62.0 versus 57.0 years; *p* < 0.001). Hypertension was more frequent in patients with SCA (64.2% versus 46.3%; *p* = 0.003), and also, as expected, they had higher systolic blood pressure (142 versus 136 mmHg; *p* = 0.003). However, the study groups were similar in terms of BMI, waist circumference, physical activity, frequency of dyslipidemia, diabetes-related characteristics (insulin treatment, glycated hemoglobin, or diabetes duration), and lipid profile.

There were no differences in 25OHD concentrations between the 2 study groups (18.4 versus 18.8 ng/mL; *p* = 0.341). Although serum calcium was similar in both groups, serum phosphate was higher in patients with SCA (3.57 versus 3.41 mg/dL; *p* = 0.026). The calcium phosphate (CaP) product was also increased in subjects with SCA (32.9 versus 31.9 mg^2^/dL^2^; *p* = 0.026). Additionally, PTH concentrations were lower in patients with SCA (42.4 versus 47.1 pg/mL; *p* = 0.027). Finally, there were no differences in terms of dietary calcium or vitamin D intake between groups. The multivariable analysis showed that there were no significant associations between female sex, 25OHD and PTH concentrations, and subclinical atherosclerosis (Table 2). However, age (OR = 1.055; 95% CI = 1.027–1.084; *p* < 0.001) and systolic blood pressure (OR = 1.016; 95% CI = 1.002–1.030; *p* = 0.028) were positively associated with the presence of carotid plaques (Table 2). Although 25OHD and PTH were not associated with the presence of plaques, we found a positive association between the CaP product and the presence of SCA (OR = 1.078; 95% CI = 1.017–1.142; *p* = 0.012) (Table 2).

Thereafter, a multivariable analysis for cIMT was also performed (Table 3). A basic age- and sex-adjusted model and the fully adjusted model are shown in Supplementary Table 1 available online at https://doi.org/10.1155/2017/3498368. Vitamin D ≥ 30 ng/mL, as compared to vitamin D < 20 ng/mL, was associated with lower common cIMT (*β* ± SE = −0.055 ± 0.024; *p* = 0.023). Age (*β* ± SE = 0.005 ± 0.0008; *p* < 0.001) and the presence of dyslipidemia (*β* ± SE = 0.042 ± 0.015; *p* = 0.006) were positively associated with common carotid intima-media thickness. There was a negative association between female sex and cIMT (*β* ± SE = −0.05 ± 0.02; *p* = 0.002). The age- and sex-adjusted model and the fully adjusted model are shown in Supplementary Table 2.

## 4. Discussion

In this study, we aimed to investigate the relationship between 25OHD and other mineral metabolism-related factors and the presence of subclinical carotid atherosclerosis, assessed by the presence of carotid plaques, in type 2 diabetic subjects. The study participants were free from chronic kidney disease without previous cardiovascular disease. Our results clearly show that increasing values of calcium phosphate product are associated with the presence of atherosclerotic carotid plaques. Concentrations of 25OHD in the higher range were negatively associated with lower cIMT in these subjects.

Existing experimental and clinical evidence points to the involvement of phosphate and calcium in the development of atherosclerosis [18, 20]. Most of the evidence in this field is restricted to the study of the role of mineral metabolism in chronic kidney failure. Actually, phosphate has been shown to be associated with subclinical atherosclerosis in patients with advanced kidney disease [31]. The dysregulation of mineral metabolism induced by hyperphosphatemia accompanying the impairment of kidney function is considered one of the main drivers of increased cardiovascular disease in these patients [18]. Different pieces of evidence point to CaP product as a potential marker of future cardiovascular events. Most of the studies addressing this issue have been performed in patients with chronic kidney disease [17, 21, 22]. CaP product is a marker of adverse outcomes in patients with peripheral artery disease under hemodialysis [17]. Additionally, future mortality in patients receiving chronic dialysis is also predicted by CaP product [22]. Further, a recent study in young subjects with chronic kidney disease showed a clear positive association of CaP product and fibroblast growth factor 23 (FGF-23) [21]; the latter is involved in vascular calcification and is associated with future cardiovascular morbidity and mortality in patients with renal insufficiency [18, 20].

Very few additional studies were done in subjects without chronic kidney disease [16, 19]. A very well-known community-based epidemiological study of cardiovascular disease in the US showed that calcium, phosphate, and CaP product were risk factors for stroke and death after more than 12 years of follow-up [16]. Further, a very large study in subjects undergoing coronary disease assessment for a variety of clinical indications, including actual cardiovascular disease events, found a clear association of calcium, phosphate, and CaP product with the presence of coronary atherosclerotic disease [19]. Unfortunately, although these studies included a low proportion of subjects with diabetes, we have not been able to identify any specific studies addressing this question in patients with T2DM. Also, none of these studies included additional markers of mineral metabolism, such as PTH and 25OHD. Thus, previous and the current findings suggest the need for further research in this specific area, especially in patients with diabetes and, in general, in subjects without chronic kidney disease.

Regarding vitamin D, our results only showed that subjects with higher 25OHD concentrations (≥30 ng/mL) had lower cIMT. It should be pointed out that cIMT reflects initial morphologic changes in the intima of the artery wall. However, the existence of a plaque indicates that the atherosclerotic process is established; actually, cardiovascular events take place due to plaque complications. In our study, the concentrations of 25OHD were not at all associated with the presence of carotid plaques. These findings may indicate that 25OHD could be a marker of future cardiovascular disease only in early stages of the atherosclerotic process in diabetes. In contrast to the current results, Chen et al. described the association of lower 25OHD levels and the presence of carotid plaques in Chinese type 2 diabetic patients [32]. Previous cross-sectional studies found an association between lower levels of 25OHD and cIMT [12, 33, 34]. In the large prospective Framingham Offspring Study, vitamin D deficiency was a predictor of cardiovascular disease only in subjects with hypertension. More importantly, in two meta-analyses, 25OHD was linked to coronary disease [14] and cardiovascular and all-cause mortality [11]. Finally, the Multi-Ethnic Study of Atherosclerosis, a large prospective cohort study, the authors observed a complete lack of association of 25OHD and PTH with baseline cIMT and plaque prevalence and progression of these markers of subclinical atherosclerosis at follow-up [15]. It must be pointed out that, from all these studies, only the one by Carrelli et al. included also the assessment of circulating calcium and phosphate; moreover, these authors observed that, apart from 25OHD, phosphate and CaP product were associated with subclinical atherosclerosis. Overall, these results give further support to the accumulating evidence of the role of mineral metabolism in the atherosclerotic pathogenetic process. However, none of these factors have been consistently shown to be predictors of future cardiovascular disease in all the study settings and populations [11]. We strongly believe that further investigations are warranted in this field. On one hand, we are in need of further experimental research. On the other hand, clinical prospective studies are also warranted. Unfortunately, very few studies have assessed all the main factors (25OHD, PHT, calcium, phosphate, and FGF-23) involved in the mineral metabolism pathway together in the same study subjects. The partial information given by a single factor does not actually provide a whole picture of this potential pathogenetic pathway contributing to atherosclerosis. We strongly believe that the assessment of the vitamin D status without the determination of other parameters of mineral metabolism must be avoided in future studies.

The current study has some limitations. First, this is a cross-sectional study that was not specifically designed to address the associations investigated in the current substudy. Additionally, this is not a population-based study, and this may limit the external validity of the current findings. Nevertheless, the characteristics of the included patients are very similar to those of the general diabetic population in our region [35]. The current study has addressed the question in a large group of well-characterized type 2 diabetic patients that included extensive work-up on mineral metabolism and subclinical carotid atherosclerosis (cIMT and plaques). Although we assessed different factors involved in the regulation of mineral metabolism, we did not assess additional important molecules that could have extended the knowledge, such as FGF-23.

## 5. Conclusions

In conclusion, the current findings indicate that the CaP product is associated with established subclinical atherosclerosis in patients with T2DM without previous cardiovascular disease or chronic kidney disease. Additionally, higher circulating 25OHD concentrations (≥30 ng/mL) are associated with lower cIMT. Given that this was a secondary analysis, the reported associations need to be confirmed in a prospectively designed study. This new research should include a comprehensive phenotyping of participants and should not focus in the study of an isolated factor of mineral metabolism but rather should assess the wide range of molecules that are involved in the complex cross-talk between parameters of mineral metabolism, that is, 25OHD, phosphate, calcium, PTH, and FGF-23.

## Supplementary Material

The information of supplementary materials are as follows: Supplementary Table 1. Multivariate analysis models for the presence of carotid plaques. Supplementary Table 2. Multivariate analysis models for common carotid intima-media thickness.

## Figures and Tables

**Table 1:** Clinical and biochemical characteristics of patients with type 2 diabetes with and without subclinical carotid atherosclerosis (SCA).

	No SCA *n* = 123	SCA *n* = 180	*p* value
Age (years)	57.0 [48.0; 64.0]	62.0 [54.0; 69.0]	<0.001
Female sex	66 (53.7)	83 (46.1)	0.241
Caucasian	114 (92.7)	175 (97.2)	0.116
Current smoking	20 (16.3)	40 (22.2)	0.258
Physical activity (minutes)	60 (50.8)	80 (47.6)	0.676
Hypertension	57 (46.3)	115 (64.2)	0.003
Systolic blood pressure (mmHg)	136 ± 17.1	142 ± 19.6	0.003
Diastolic blood pressure (mmHg)	77.5 ± 10.6	76.6 ± 10.9	0.504
Dyslipidemia	51 (41.5)	89 (49.7)	0.195
Insulin treatment	39 (31.7)	62 (34.4)	0.710
Disease duration (years)	7.00 [4.00; 12.5]	8.50 [4.00; 15.0]	0.195
Body mass index (kg/m^2^)	30.6 [28.3; 34.7]	30.6 [27.7; 34.4]	0.280
Waist circumference (cm)	105 ± 11.8	105 ± 10.9	0.903
Season			0.690
Spring	30 (24.4)	53 (29.4)	
Summer	44 (35.8)	59 (32.8)	
Autumn	32 (26.0)	40 (22.2)	
Winter	17 (13.8)	28 (15.6)	
HbA1c (%)	7.55 [6.60; 8.60]	7.60 [6.90; 8.40]	0.970
Total cholesterol (mg/dL)	183 [162; 210]	182 [164; 204]	0.969
HDL-c (mg/dL)	46.0 [40.0; 57.0]	49.0 [42.0; 60.0]	0.065
LDL-c (mg/dL)	107 [86.8; 130]	107 [89.2; 127]	0.919
Triglycerides (mg/dL)	122 [85.0; 181]	110 [82.8; 155]	0.105
Serum creatinine (mg/dL)	0.77 [0.67; 0.90]	0.80 [0.69; 0.93]	0.480
Urinary albumin/creatinine ratio (mg/g)	7.10 [3.60; 15.5]	8.43 [3.97; 20.3]	0.318
25OHD (ng/mL)	18.8 [13.2; 26.2]	18.4 [13.3; 24.2]	0.341
Vitamin D levels			0.530
<20 ng/mL	65 (52.8)	106 (58.9)	
20–<30 ng/mL	42 (34.1)	56 (31.1)	
≥30 ng/mL	16 (13.0)	18 (10.0)	
Dietary vitamin D intake (*μ*g/day)	4.47 [2.93; 5.70]	3.88 [2.92; 5.05]	0.181
Dietary calcium intake (mg/day)	1182 [866; 1467]	1116 [811; 1379]	0.155
Parathormone (pg/mL)	47.1 [36.8; 56.6]	42.4 [33.9; 52.3]	0.027
Calcium (mg/dL)	9.25 [9.01; 9.42]	9.29 [9.06; 9.48]	0.312
Phosphate (mg/dL)	3.41 [3.13; 3.76]	3.57 [3.28; 3.84]	0.026
Calcium phosphate product (mg^2^/dL^2^)	31.9 [29.0; 35.2]	32.9 [30.3; 36.2]	0.026
cIMT (mm)	0.73 [0.66; 0.80]	0.83 [0.73; 0.91]	<0.001

Values are mean ± SD, *n* (%) or median [interquartile range]. HbA1c: glycated hemoglobin; HDL-c: high-density lipoprotein cholesterol; LDL-c; low-density lipoprotein cholesterol; 25OHD: 25-hydroxyvitamin D_3,_ cIMT: common carotid intima-media thickness.

**Table 2:** Multivariable analysis for the presence of carotid plaques.

	OR (95% CI)	*p* value
Age (years)	1.055 (1.027–1.084)	<0.001
Female sex	0.661 (0.388–1.125)	0.127
Systolic blood pressure (mmHg)	1.016 (1.002–1.030)	0.028
Calcium phosphate product (mg^2^/dL^2^)	1.078 (1.017–1.142)	0.012
PTH (pg/mL)	0.989 (0.974–1.004)	0.151
25OHD (ng/mL)	0.991 (0.965–1.018)	0.530

Adjusted OR and their 95% CI estimated according to multivariable logistic regression model; Hosmer-Lemeshow test, *p* value = 0.837.

**Table 3:** Multivariable analysis for common carotid intima-media thickness.

Coefficients	Estimate (standard error)	*p* value
Age (years)	0.0047155 (0.0007817)	<0.001
Female sex	−0.0479803 (0.0150747)	0.002
Dyslipidemia	0.0421709 (0.0150771)	0.006
Vitamin D (20–30) versus vitamin D < 20 (ng/mL)	−0.0189342 (0.0165022)	0.252
Vitamin D ≥ 30 versus vitamin D < 20 (ng/mL)	−0.0549896 (0.0240509)	0.023

Adjusted estimates and standard error computed according to multivariable linear regression model; Multiple R-squared, 0.1778; adjusted R-squared, 0.1627; Kolmogorov-Smirnov test, *p* value = 0.200.

## Abbreviations

CaP product: Calcium phosphate product
cIMT: Carotid intima-media thickness
FGF-23: Fibroblast growth factor 23
HbA1c: Glycated hemoglobin
HDL-c: High-density lipoprotein cholesterol
LDL-c: Low-density lipoprotein cholesterol
25OHD: 25-Hydroxyvitamin D_3_
PTH: Parathormone
SCA: Subclinical carotid atherosclerosis
T2DM: Type 2 diabetes mellitus.
