# Supplementary material for: Calcium Phosphate Product Is Associated with Subclinical Carotid Atherosclerosis in Type 2 Diabetes
Source: J Diabetes Res. 2017 Aug 3;2017:3498368. doi: 10.1155/2017/3498368 (PMC5559910; doi:10.1155/2017/3498368)
Supplement: Supplementary file 1 — The information of supplementary materials are as follows: Supplementary Table 1. Multivariate analysis models for the presence of carotid plaques. Supplementary Table 2. Multivariate analysis models for common carotid intima-media thickness. [file 3498368.f1.docx]

| **Supplementary Table1. Multivariate analysis models for the presence of carotid plaques** | | | | | | | | | | | | |
| --- | --- | --- | --- | --- | --- | --- | --- | --- | --- | --- | --- | --- |
|  | Fully adjusted model | | | | Sex- and age-adjusted model | | | | Reduced model | | | |
| Variable | p-value | OR | 95%CI | | p-value | OR | 95%CI | | p-value | OR | 95%CI | |
|  |  |  |  |  |  |  |  |  |  |  |  |  |
| Age (years) | <0.001 | 1.064 | 1.030 | 1.099 | <0.001 | 1.058 | 1.031 | 1.086 | <0.001 | 1.055 | 1.027 | 1.084 |
| Female sex | 0.08 | 0.522 | 0.252 | 1.082 | 0.019 | 0.551 | 0.334 | 0.908 | 0.127 | 0.661 | 0.388 | 1.125 |
| 25OHD (ng/mL) | 0.761 | 0.996 | 0.967 | 1.025 | 0.541 | 0.992 | 0.966 | 1.018 | 0.530 | 0.991 | 0.965 | 1.018 |
| Calcium phosphate product (mg^2^/dL^2^) | 0.022 | 1.079 | 1.011 | 1.151 | 0.002 | 1.09 | 1.032 | 1.151 | 0.012 | 1.078 | 1.017 | 1.142 |
| PTH (pg/mL) | 0.236 | 0.99 | 0.974 | 1.006 |  |  |  |  | 0.151 | 0.989 | 0.974 | 1.004 |
| Systolic blood pressure (mmHg) | 0.017 | 1.023 | 1.004 | 1.042 |  |  |  |  | 0.028 | 1.016 | 1.002 | 1.03 |
| Diastolic blood pressure (mmHg) | 0.378 | 0.987 | 0.957 | 1.017 |  |  |  |  |  |  |  |  |
| Current smoking | 0.081 | 1.848 | 0.928 | 3.68 |  |  |  |  |  |  |  |  |
| Physical activity | 0.378 | 0.789 | 0.466 | 1.337 |  |  |  |  |  |  |  |  |
| Dyslipidemia | 0.568 | 1.168 | 0.686 | 1.989 |  |  |  |  |  |  |  |  |
| Body mass index (kg/m^2^) | 0.769 | 0.992 | 0.938 | 1.048 |  |  |  |  |  |  |  |  |
| Disease duration (years) | 0.659 | 1.008 | 0.972 | 1.046 |  |  |  |  |  |  |  |  |
| HbA1c (%) | 0.095 | 0.835 | 0.675 | 1.032 |  |  |  |  |  |  |  |  |
| Creatinine (mg/dL) | 0.17 | 0.256 | 0.037 | 1.788 |  |  |  |  |  |  |  |  |
| Urinary albumin/ creatinine ratio (mg/g) | 0.847 | 0.999 | 0.993 | 1.006 |  |  |  |  |  |  |  |  |
| Hosmer-Lemeshow test p-value | 0.678 |  |  |  | 0.655 |  |  |  | 0.837 |  |  |  |

95%CI: 95% Confidence Interval

| **Supplementary Table 2. Multivariate analysis models for common carotid intima-media thickness** | | | | | | | | | | |
| --- | --- | --- | --- | --- | --- | --- | --- | --- | --- | --- |
|  | Fully adjusted model | | | Sex- and age-adjusted model | | | Reduced model | |  |  |
| Variable | | Coefficient | SE | p-value | Coefficient | SE | p-value | Coefficient | SE | p-value |
| Age (years) | | 0.005 | 0.001 | <0.001 | 0.005 | 0.001 | <0.001 | 0.005 | 0.001 | <0.001 |
| Female sex | | -0.075 | 0.022 | 0.001 | -0.050 | 0.016 | 0.001 | -0.048 | 0.015 | 0.002 |
| Vitamin D (20 - <30) vs. <20 (ng/mL) | | -0.002 | 0.017 | 0.888 | -0.020 | 0.017 | 0.225 | -0.019 | 0.017 | 0.252 |
| Vitamin D ≥30 vs. <20 (ng/mL) | | -0.051 | 0.026 | 0.046 | -0.061 | 0.024 | 0.013 | -0.055 | 0.024 | 0.023 |
| Calcium phosphate product (mg^2^/dL^2^) | | 0.002 | 0.002 | 0.215 | 0.002 | 0.002 | 0.158 |  |  |  |
| Dyslipidemia | | 0.034 | 0.016 | 0.033 |  |  |  | 0.042 | 0.015 | 0.006 |
| Current smoking | | 0.030 | 0.020 | 0.138 |  |  |  |  |  |  |
| Physical activity | | -0.013 | 0.016 | 0.394 |  |  |  |  |  |  |
| Systolic blood pressure (mmHg) | | 0.001 | 0.001 | 0.097 |  |  |  |  |  |  |
| Diastolic blood pressure (mmHg) | | -0.001 | 0.001 | 0.315 |  |  |  |  |  |  |
| Body mass index (kg/m^2^) | | 0.001 | 0.002 | 0.568 |  |  |  |  |  |  |
| Disease duration (years) | | 0.000 | 0.001 | 0.724 |  |  |  |  |  |  |
| HbA1c (%) | | 0.000 | 0.006 | 0.980 |  |  |  |  |  |  |
| Serum creatinine (mg/dL) | | -0.080 | 0.057 | 0.162 |  |  |  |  |  |  |
| Urinary albumin/ creatinine ratio(mg/g) | | 0.000 | 0.000 | 0.441 |  |  |  |  |  |  |
| PTH (pg/mL) | | 0.001 | 0.001 | 0.237 |  |  |  |  |  |  |
| Model diagnostics | |  |  |  |  |  |  |  |  |  |
| Multiple R-squared | |  |  | 0.227 |  |  | 0.160 |  |  | 0.178 |
| Adjusted R-squared | |  |  | 0.176 |  |  | 0.145 |  |  | 0.163 |
| Kolmogorov-Smirnov test p-value | |  |  | 0.200 |  |  | 0.086 |  |  | 0.200 |

SE: Standard Error
